# *Bacillus velezensis* M4 from Northeast Chinese Soybean Paste Combines Nattokinase and Antibacterial Activities

**DOI:** 10.3390/foods15091553

**Published:** 2026-04-30

**Authors:** Yin Feng, Yuexin Gao, Linxi Wang, Bo Nan, Jingsheng Liu, Yuhua Wang

**Affiliations:** 1 College of Food Science and Engineering, Jilin Agricultural University, Changchun 130033, China; fengyin1123@sina.com (Y.F.); gauyuexin@163.com (Y.G.); 18392583879@163.com (L.W.); nanbo@jlau.edu.cn (B.N.); liujingsheng@jlau.edu.cn (J.L.); 2 School of Life Science, Changchun SCI-TECH University, Changchun 130600, China; 3 Jilin Province Innovation Center for Food Biological Manufacture, Jilin Agricultural University, Changchun 130033, China; 4 National Processing Laboratory for Soybean Industry and Technology, Changchun 130033, China; 5 National Engineering Laboratory for Wheat and Corn Deep Processing, Changchun 130033, China

**Keywords:** *Bacillus velezensis*, nattokinase, probiotic, whole-genome sequencing

## Abstract

A bacterial strain M4 exhibiting high nattokinase (NK) activity and favorable antibacterial properties was isolated from fermented soybean paste in Northeast China. Based on morphological observation, physiological and biochemical characterization, 16S rDNA sequence analysis, and whole-genome sequencing, the strain was identified as *Bacillus velezensis*. Its probiotic potential and safety were systematically evaluated using a combination of in vitro assays and genome mining. Genomic analysis revealed that M4 possessed a complete genome consisting of a single circular chromosome of 4,473,838 bp with a GC content of 46.94%, encoding 4516 predicted proteins. Functional domain annotation identified four proteins (XLQ58132.1, XLQ58158.1, XLQ59409.1, and XLQ59873.1) containing both the Peptidase inhibitor I9 and Peptidase S8 domains, confirming the presence of the typical molecular signature of NK. Furthermore, the genome harbored 132 genes encoding carbohydrate-active enzymes, 37 biosynthetic gene clusters, and 142 genes encoding proteolytic enzymes. Comparative genomic analysis revealed a close phylogenetic relationship with other *B. velezensis* strains and identified 98 strain-specific genes. Safety assessment demonstrated that M4 exhibited no hemolytic activity, was susceptible to eight commonly tested antibiotics, and lacked genes encoding high-risk virulence factors. Probiotic characterization indicated that M4 exhibited certain levels of gastrointestinal tolerance, acid resistance, bile salt resistance, antioxidant activity, and antibacterial properties. In conclusion, *B. velezensis* M4 shows potential for development as a functional strain.

## 1. Introduction

Cardiovascular disease remains one of the leading causes of mortality worldwide, with thrombosis representing a critical pathological mechanism underlying its progression. Nattokinase (NK), a bacterial-derived serine protease with potent fibrinolytic activity, has emerged as a promising thrombolytic agent due to its ability to directly hydrolyze fibrin, oral bioavailability, and favorable safety profile [1,2]. Concurrently, increasing attention has been directed toward intestinal health, wherein strains exhibiting antibacterial activity offer unique advantages in modulating gut microbiota and inhibiting pathogen colonization [3,4]. However, most currently available strains display limited functional diversity, and isolates capable of simultaneously producing NK and exerting broad-spectrum antibacterial effects remain scarce. Therefore, the screening of strains with multiple functional characteristics holds significant importance for the development of functional microorganisms and represents a key research direction in this field.

In recent years, *Bacillus velezensis* has received extensive attention as a potential probiotic in the food, livestock, and agricultural sectors. Studies have shown that *B. velezensis* can produce a variety of antimicrobial substances (e.g., lipopeptides and polyketides) and exhibits beneficial effects in animal production, including improved growth performance, enhanced immune function, and inhibition of pathogenic bacteria [5,6,7,8,9]. However, most of the *B. velezensis* probiotic strains reported to date have focused primarily on a single function, such as antibacterial activity or enzyme production. Although some *B. velezensis* strains (e.g., BV702, BV379, BVQ121) have been reported to produce NK or exhibit probiotic properties [8,10,11,12,13], strains with both high NK activity and broad-spectrum antibacterial activity are still very rare.

Moreover, existing studies have largely concentrated on phenotypic evaluation, lacking systematic analysis of the functional genome. In particular, research linking phenotypic traits to genomic features (e.g., biosynthetic gene clusters of secondary metabolites, strain-specific genes) is still very limited. Therefore, the isolation of a *B. velezensis* strain that combines NK activity with antibacterial function, coupled with whole-genome analysis to elucidate its molecular basis, will help deepen the understanding of the multifunctional mechanisms of this strain and provide a theoretical basis and strain resources for the development of novel probiotics with composite functional characteristics.

In this study, strain M4 was isolated from Northeast Chinese soybean paste and characterized by high NK activity and favorable antibacterial activity. The strain was identified as *B. velezensis* based on morphological, physiological, and biochemical characteristics, 16S rDNA sequencing, and whole-genome sequencing. The genome of *B. velezensis* M4 was annotated using multiple databases, and comparative genomic analysis was performed against previously reported *B. velezensis* strains to reveal distinctive genomic features. Furthermore, the probiotic properties and safety of *B. velezensis* M4 were evaluated through in vitro experiments combined with genome mining. Assessed probiotic characteristics included gastrointestinal tolerance, bile salt resistance, antioxidant capacity, and antibacterial activity, while safety evaluation encompassed hemolytic activity, antibiotic susceptibility testing, and genomic analysis of antibiotic resistance genes (ARGs) and virulence factors (VFs). This study establishes a foundation for the potential application of *B. velezensis* M4 as a fermented probiotic.

## 2. Materials and Methods

### 2.1. Materials and Chemical Reagents

The Northeast Chinese soybean paste was sourced from Jilin Tianyequan Brewing Co., Ltd. (Changchun, China). The bacterial strains, comprising *E. coli*, *S. aureus*, and *Salmonella*, were acquired from Luwei Technology Co., Ltd. in Shanghai, China. Concentrated hydrochloric acid, hydrochloric acid, disodium hydrogen phosphate, glucose, absolute ethanol, agar, sodium chloride, magnesium sulfate, ox bile salt, and beef extract powder were procured from Sinopharm Chemical Reagent Co., Ltd. (Shanghai, China). Peptone and yeast extract powder were obtained from Oxoid Ltd. in the Basingstoke, UK. Pepsin (10,000 U/g), trypsin (250 U/mg), DPPH, and ABTS reagents were sourced from Shanghai Yuanye Bio-Technology Co., Ltd. (Shanghai, China). Antibiotic susceptibility test discs were purchased from Biocomma Limited (Changde, China).

### 2.2. Screening, Isolation, and Purification of Strains with High NK Activity and Antibacterial Activity

1. Primary Screening

One gram of naturally fermented soybean paste was weighed and mixed with sterile water at a mass ratio of 1:9, followed by vortexing. The mixture was then placed in a boiling water bath for 10 min and cooled to room temperature. The treated sample was serially diluted to appropriate concentrations, and 0.1 mL of each dilution was spread-plated onto casein solid agar medium. After incubation at 37 °C for 48 h, 20 colonies exhibiting relatively large hydrolysis zones were selected for Gram staining and microscopic examination. Gram-positive strains were subsequently transferred to fresh LB solid medium using the streak plate method and incubated at 37 °C for 24 h. This purification procedure was repeated 3–4 times until colonies with uniform morphology were obtained, indicating successful strain isolation.

2. Secondary Screening

The initially screened strains were inoculated into a liquid fermentation medium (yeast extract 5 g/L, peptone 10 g/L, glucose 10 g/L, Na_2_HPO_4_ 4 g/L, and MgSO_4_ 0.2 g/L) specifically designed for *Bacillus* species and cultured at 37 °C with shaking at 180 r/min for 24 h. NK activity of these strains was determined following the method described by Feng et al. [12]. Meanwhile, antibacterial activity was assessed against Gram-negative (*E. coli*, *Salmonella* spp.) and Gram-positive (*S. aureus*) indicator strains. Specifically, the fermentation broth of the preliminary screened strains was adjusted to a bacterial concentration of 10^8^ CFU/mL. Aliquots of the fermentation broth were then added into Oxford cups placed on LB agar plates previously inoculated with the respective indicator bacteria. After incubation at 37 °C for 24 h, the formation of inhibition zones was observed. Strains exhibiting both high NK activity and favorable antibacterial properties were further purified by the streak plate method (The screening results are shown in Supplementary Material S1: Table S1). This purification process was repeated 3–4 times until morphologically homogeneous single colonies were obtained. The resulting selected target strain, designated as M4, was preserved at −80 °C for subsequent experiments. M4 was then inoculated onto LB agar plates and incubated at 37 °C for 24 h. Its colony morphology was observed, and Gram staining was performed to examine its cellular morphology. A single colony of M4 was transferred into 3 mL of sterile physiological saline for physiological and biochemical tests, including assays for NaCl tolerance, citrate utilization, starch hydrolysis, casein degradation, and the Voges–Proskauer test. Each physiological and biochemical test was independently repeated three times. The cellulase activity was determined by using the DNS method [14]. The protease activity was measured in accordance with the method described by Xu et al. [15]. The α-amylase activity was evaluated based on the method outlined by Ye et al. [16] to further characterize the strain.

### 2.3. 16S rDNA Sequencing of the M4 Strain

For molecular identification, the strains activated on LB solid medium for 12 h were inoculated into LB liquid medium and cultured at 37 °C with shaking at 180 rpm for 12–16 h to obtain a bacterial suspension (BS). The bacterial cells were collected, and the genomic DNA was extracted as per the instructions of the kit (CW0552; Jiangsu Kangwei Century Biotechnology Co., Ltd., Taizhou, China). The extracted genomic DNA was then used as the template for amplification with universal bacterial 16S rDNA primers 27F (5′-AGAGTTTGATCCTGGCTCAG-3′) and 1492R (5′-ACGGCTACCTTGTTACGCTT-3′), which were synthesized by Sangon Biotech Co., Ltd. (Shanghai, China). The 25-μL amplification system contained 2 μL of genomic DNA template, 1 μL of each primer (10 μM), 12.5 μL of 2× EasyTaq^®^ PCR SuperMix (AS111; TransGen Biotech, Beijing, China), and 8.5 μL of ddH_2_O. The amplification program was as follows: initial denaturation at 95 °C for 5 min; 34 cycles of denaturation at 94 °C for 30 s, annealing at 50 °C for 30 s, and extension at 72 °C for 90 s; with a final extension at 72 °C for 10 min. The PCR products were purified and sequenced by Sangon Biotech Co., Ltd. The obtained sequences were compared using BLAST v2.16.0 on the NCBI website and subjected to clustering analysis using MEGA 6.0 software. A phylogenetic tree was constructed using the neighbor-joining method, and bootstrap analysis was performed with 1000 replicates.

### 2.4. Whole-Genome Sequencing, Assembly, and Annotation of M4 Strain

A single colony of M4 was inoculated into LB medium and cultivated at 37 °C with shaking at 180 rpm until OD_600_ reached 0.6. Cells were harvested by centrifugation, washed with PBS, and frozen in liquid nitrogen for whole-genome sequencing (Personal Biotechnology Co., Ltd., Shanghai, China). Genomic DNA was extracted using the cetyltrimethylammonium bromide method, quantified fluorometrically, and assessed for integrity by 1% agarose gel electrophoresis. Sequencing libraries were prepared for Illumina NovaSeq (second-generation) and PacBio Sequel (third-generation) platforms, yielding 1.00 Gb cleaned data (224× coverage) and 1.20 Gb long reads (269× coverage), respectively. PacBio reads were assembled using Unicycler v0.5.0, producing a single contig with an N50 of 4,473,838 bp. The assembly was polished with Pilon v1.24 using Illumina short reads and assessed for completeness using CheckM v1.1.6 (99.81% complete). Gene prediction was performed with GeneMarkS v4.28, and functional annotation was conducted against GO, COG, and KEGG databases. Non-coding RNAs were predicted using tRNAscan-SE v2.0, RNAmmer v1.2, and Rfam v15.1. Carbohydrate-active enzymes and biosynthetic gene clusters were identified using CAZyme (run_dbcan (v5.0.6)) and antiSMASH v7.1.0, respectively. Proteolytic enzymes were annotated using MEROPS v8.5 [17]. For putative NK gene screening, protein-coding genes predicted by Prodigal were analyzed against the Pfam database (http://pfam.xfam.org) using HMMER v3.4. Candidates were selected based on the simultaneous presence of Peptidase inhibitor I9 (PF05922) and Peptidase S8 (PF00082) domains, with an E-value threshold of <0.01. A circular genome map was generated using CGView v1.0 [18].

### 2.5. Comparative Genomic Analysis

#### 2.5.1. Genomic Data Acquisition

A total of 19 *B. velezensis* strains (Supplementary Material S1: Table S6) were downloaded from NCBI, and regional and host information were collected for each genome, along with CheckM genome quality assessment results.

#### 2.5.2. Collinearity Analysis

The M4 genome was used as the target, with the other 19 genomes serving as references. The evolutionary distances were assessed through collinearity analysis to elucidate the phylogenetic relationships between the target and reference species. Whole-genome alignment was performed using MUMmer (v3.1) [19] to identify large-scale collinear blocks. Subsequently, SyRI (v1.4) [20] was employed to compare these aligned regions, confirm local synteny, and identify genomic variations, including translocations, inversions, and combined translocation-inversion events. The genomic coordinates of all aligned blocks were then recorded.

#### 2.5.3. SNP/InDel/SV Analysis

SNPs were identified by aligning the assembled genomes using MUMmer (v3.1). The identified SNPs were annotated based on their genomic context relative to the gene features. Small insertions and deletions <50 bp were detected using MUMmer. For larger structural variants (≥50 bp), including insertions, deletions, inversions, and translocations, the software SyRI (v1.4) was used for detection.

#### 2.5.4. Gene Family Analysis

The gene families were identified based on the bidirectional best-hit (BBH) criterion, requiring at least 40% amino acid identity over 80% of the length of the shorter protein. All-versus-all protein sequence alignment was performed using DIAMOND v2.0.7. Subsequently, OrthoMCL v2.0 was employed to cluster these sequences into homologous groups [21]. The distribution of species across these protein clusters was then statistically analyzed.

#### 2.5.5. Core-Pan Genome Analysis

The genome sequences of M4 and 19 other related *B. velezensis* strains were aligned using Mugsy (v1.2.3). Pan-genome and core-genome analyses were conducted using the Prokaryotic Pan-genome Analysis pipeline. The gene families were constructed to define the pan-genome (total gene repertoire) and core-genome (shared genes). The pan-genome and core-genome curves were fitted based on Heap’s law and an exponential decay model, respectively [22].

#### 2.5.6. Phylogenetic Tree Analysis

A phylogenetic tree was inferred using the maximum likelihood method to elucidate the evolutionary relationships among the analyzed genomes. Single-copy orthologous genes, identified from the gene family clustering analysis, were extracted. Multiple sequence alignment of these protein sequences was performed with MUSCLE [23], followed by alignment refinement with Gblocks to remove poorly aligned regions [24]. The best-fit substitution model was automatically selected, and the phylogenetic tree was constructed using IQ-TREE v1.0 [25].

#### 2.5.7. ANI/AAI Analysis

The ANI analysis between the M4 genome and each reference genome was calculated using PyANI (v0.2.7) [26]. In addition, the AAI analysis was determined using CompareM (v0.0.23) based on the predicted protein sequences (https://github.com/dparks1134/CompareM (accessed on 30 April 2025). 

### 2.6. Safety Assessment

#### 2.6.1. Hemolysis Assay

The frozen stock strains were reactivated for at least two successive passages. A single, well-isolated colony was selected and streaked onto freshly prepared blood agar plates (containing 5% sterile defibrinated sheep blood). After inoculation, the plates were incubated aerobically at 37 °C for 24 h. Following incubation, the plates were examined macroscopically for the presence or absence of a distinct hemolytic zone surrounding each colony [27].

#### 2.6.2. Antibiotic Susceptibility Testing

Antimicrobial susceptibility was determined by the Kirby–Bauer disk-diffusion method [28]. The M4 was cultured in LB broth at 37 °C with shaking at 200 rpm until reaching the mid-logarithmic phase. A 100-μL aliquot of the suspension was then spread uniformly onto LB agar plates to obtain a confluent lawn. Sixteen antibiotic disks, including erythromycin, chloramphenicol, tetracycline, vancomycin, kanamycin, clindamycin, streptomycin, gentamicin, ampicillin, cefotaxime, amikacin, florfenicol, ciprofloxacin, rifampicin, lincomycin, and minocycline, were aseptically applied onto the inoculated surface. The plates were then incubated aerobically at 37 °C for 24 h. After incubation, the diameter of each inhibition zone was measured to the nearest millimeter using a digital caliper. The results were interpreted in accordance with the Clinical and Laboratory Standards Institute breakpoints, and the isolate’s resistance profile was established accordingly.

#### 2.6.3. Identifying Safety-Related Genes from the Complete Genome Sequence of M4

ARGs were annotated using AMRFinderPlus v4.0.23 [29], and VFs were identified using the Virulence Factor Database (VFDB) (http://www.ncbi.nlm.nih.gov/pubmed/39470738 (accessed on 6 May 2025)). 

### 2.7. Probiotic Properties Assessment

#### 2.7.1. Bile Salt Tolerance

To assess bile salt tolerance, the M4 strain was inoculated into LB broth supplemented with 0.2% to 1.0% (*w*/*v*) bile salts. The cultures were incubated at 37 °C with orbital shaking at 160 rpm. Optical density at 600 nm (OD_600_) was recorded every 2 h for a total of 24 h, and the growth curves were constructed from the resulting data [30].

#### 2.7.2. Thermotolerance Assessment

A single colony of M4 was transferred into LB broth and cultivated at 37 °C for 24 h to obtain a stationary-phase culture. Then, 1 mL aliquots of this culture were dispensed into sterile tubes and exposed to 40 °C, 50 °C, or 60 °C in a circulating water bath with shaking at 160 rpm; an unheated control was maintained at 37 °C. At 30 min intervals, the samples were serially diluted and plated onto LB agar for viable counts [22]. The survival rate was calculated as follows:

(1) $$I\%=(A_{t}/A_{0})\times 100\%$$

where A_0_ represents the initial viable count at 37 °C, and A_t_ is the viable count after heat treatment.

#### 2.7.3. Acid Tolerance

LB broth was adjusted to pH 2.0, 3.0, and 4.0 with 1 mol/L HCl or NaOH. The isolated strain was inoculated into each pH-modified medium and incubated at 37 °C under continuous orbital shaking (160 rpm). At 30 min intervals, the cultures were serially diluted and plated onto LB agar to enumerate viable cells. Survival percentage was calculated as specified in Section 2.7.2.

#### 2.7.4. Simulated Gastric Fluid (SGF) Tolerance

Sterile 0.85% (*w*/*v*) saline was adjusted to pH 2.5 with 1 mol/L HCl and supplemented with 0.5% (*w*/*v*) pepsin to prepare SGF. An overnight culture (24 h) was inoculated into SGF at a 1:10 (*v*/*v*) ratio, followed by incubation at 37 °C in a water bath for 3 h. The samples were taken every 30 min, serially diluted, and plated on LB agar for viable counts. The survival percentage was calculated as described in Section 2.7.2.

#### 2.7.5. Simulated Intestinal Fluid (SIF) Tolerance

SIF (pH 6.8) was prepared by dissolving 1% (*w*/*v*) trypsin in 100 mL phosphate-buffered saline. A 24 h culture was added to SIF at a 1:10 (*v*/*v*) ratio and incubated at 37 °C for 3 h. Every 30 min, aliquots were serially diluted and enumerated on LB agar. The survival rate was calculated as outlined in Section 2.7.2.

#### 2.7.6. Antioxidant Activity

The strain was inoculated (1%, *v*/*v*) into 50 mL of LB broth in Erlenmeyer flasks and cultivated at 37 °C with orbital shaking at 180 rpm for 24 h. The resulting culture was centrifuged at 12,000× *g* and 4 °C for 10 min to separate cells from the cell-free supernatant (FS). The pellet was washed twice with sterile 0.85% (*w*/*v*) saline and re-suspended in the same solution to obtain a BS with a final concentration of approximately 1 × 10^9^ CFU/mL. The capacities for DPPH- and ABTS-radical scavenging, as well as FRAP, were determined according to the protocols described by Qiu et al. [31].

#### 2.7.7. Antimicrobial Activity

Antimicrobial activity was evaluated using the Oxford cup method. Briefly, 200 µL of indicator bacterial suspension (adjusted to 10^6^–10^7^ CFU/mL) was spread uniformly onto LB agar plates. After the surface had dried, sterile Oxford cups (6 mm inner diameter) were aseptically placed on the agar, and each cup was filled with 200 µL of fermentation supernatant (FS) of strain M4. Cups filled with an equal volume of sterile LB broth served as controls. The plates were incubated at 37 °C for 24 h, and the diameter of the inhibition zone was measured to the nearest 0.1 mm using a vernier caliper. All experiments were performed in three independent replicates, each with three technical replicates. Meanwhile, bacterial growth inhibition was assessed by growth curve analysis. The target indicator strains were inoculated into liquid medium and cultured to the mid-logarithmic phase. The cultures were then diluted to a concentration of 1 × 10^6^–1 × 10^7^ CFU/mL. A 50 µL aliquot of this bacterial suspension was added to 1 mL of LB medium. Then, 0.4 mL of FS was introduced to constitute the treatment group. For the control group, an equal volume (0.4 mL) of fresh LB medium was added instead of FS. The cultures were incubated statically at 37 °C, and the optical density at 600 nm (OD_600_) was monitored at 2 h intervals for 24 h to construct the growth curves. Each growth curve experiment was independently repeated three times, and the data are presented as mean ± standard deviation [31].

### 2.8. Statistical Analysis

Each set of data was measured thrice, and the results were presented as the mean ±standard deviation. The experimental data were analyzed using SPSS 26. Duncan’s test was employed to analyze the significant differences among the data, with statistical significance defined as *p* < 0.05. The figures were generated using Origin 2021.

## 3. Results and Discussion

### 3.1. Isolation and Identification of M4 Strain

**Figure 1:**
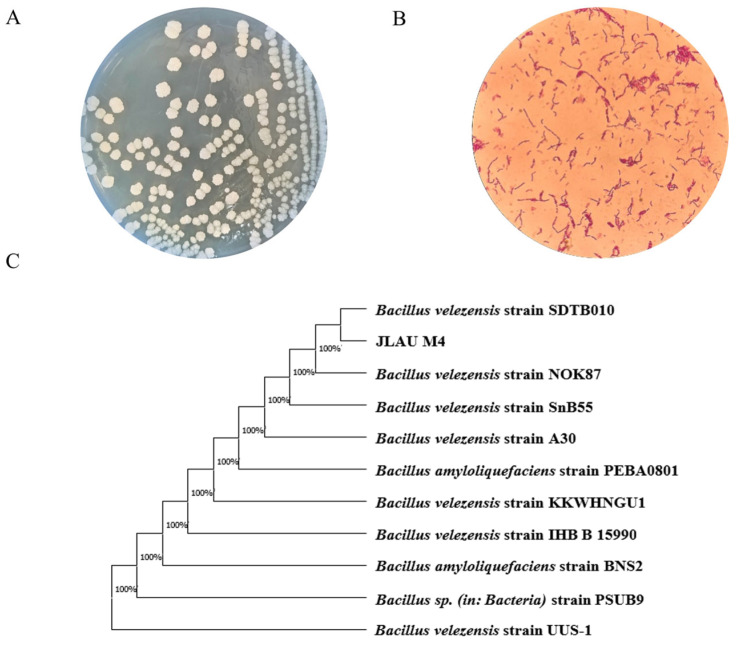
Morphological observation, molecular biological identification. (**A**) Morphological characteristics of colonies. (**B**) Gram staining result of M4 strain. (**C**) Phylogenetic tree based on 16S rDNA. The complete genome of *B. velezensis* M4.

Among the strains isolated from Northeast Chinese soybean paste, M4 exhibited a NK activity of 318.96 U/g and showed significant inhibitory effects against *E. coli*, *S. aureus*, and *Salmonella* (Supplementary Material S1: Table S1). Owing to its high NK activity and strong antibacterial properties, M4 was selected for further investigation. Colonies of M4 appeared white and round, with irregular edges and a wrinkled surface. The cells were Gram-positive, rod-shaped, and spore-forming. Physiologically, the strain tested positive for starch and casein hydrolysis but negative for citrate utilization. It could utilize various carbon sources such as D-glucose, L-rhamnose, and D-mannitol, and could grow at pH 6.94 and in 14% NaCl (Table 1). Molecular identification involved sequencing the 16S rDNA gene. Phylogenetic analysis using MEGA X, based on sequence alignment against the NCBI database, indicated that M4 was most similar to *B. velezensis*. Therefore, considering its morphological, physiological, biochemical, and molecular features, M4 was identified as *B. velezensis* (Figure 1).

### 3.2. Characterization of the Genome of Strain M4

**Figure 2:**
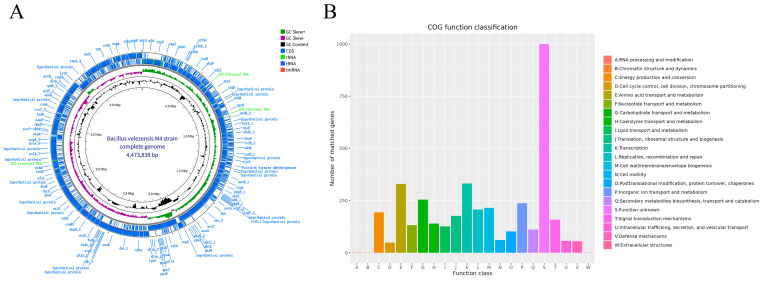
Circular map of the complete genome of *B. velezensis* M4 and COG functional classification of its protein-coding genes. (**A**) The complete genome of strain M4. (**B**) the COG functional classification results of the protein-coding genes of the M4 strain.

The M4 genome was fully sequenced and assembled, revealing a single circular chromosome of 4,473,838 bp (Figure 2A). It had a GC content of 46.94% and included 4516 coding sequences. These coding genes totaled 3,960,789 bp, accounting for 88.54% of the genome, with an average gene length of 878.67 bp. M4 also contained 86 tRNA genes, 27 rRNA genes (including 9 copies each of 5S, 16S, and 23S rRNA genes), and 93 ncRNA genes (refer to Supplementary Material S1: Tables S2 and S3). The genome sequence and annotation details are available in the NCBI database under accession number GCA_046118675.1.

### 3.3. Functional Annotation Results of the Genome of Strain M4

**Figure 3:**
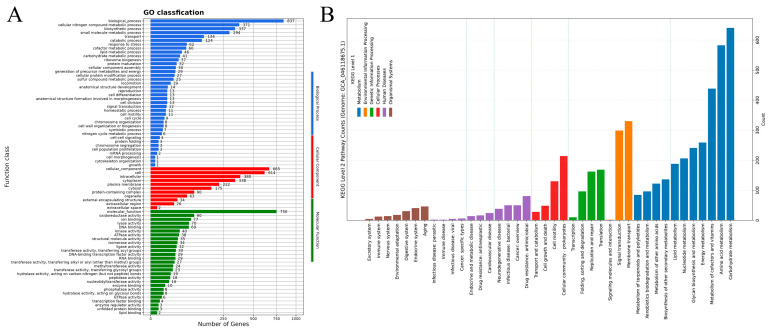
KEGG and GO annotations. (**A**) KEGG pathway classification of protein-coding genes; (**B**) GO functional classification of protein-coding genes of strain M4.

As illustrated in Figure 3A, 4926 genes were annotated in the KEGG database, divided into 6 major categories and 41 subcategories based on function. The most abundant were genes linked to protein families (1443), mainly involved in genetic information processing (593), signaling and cellular processes (546), and metabolism (304). Next were metabolism-related genes, totaling 1365, primarily associated with 11 metabolic pathways, including carbohydrate metabolism (373 genes) and amino acid metabolism (289). The GO annotation results for the M4 genome are depicted in Figure 3B. GO annotation clarifies gene roles in biological processes, cellular components, and molecular functions. This process assigned functions to 1066 protein-coding genes, producing 2661 annotations for biological processes, 2609 for cellular components, and 1494 for molecular functions (Figure 3B). The COG database, maintained by NCBI, classifies and annotates prokaryotic proteins. Upon comparing the M4 coding DNA sequence with COG, 3943 protein-coding genes were identified, representing 85.00% of all genes, grouped into 22 functional classes. Of these, 1000 genes had unknown functions, representing 25.36% of the annotated genes, highlighting the strain’s potential [22]. Additionally, 332 genes were involved in transcription, accounting for 8.42% of the annotated genes. There were 330 genes related to amino acid transport and metabolism, accounting for 8.37%, and 255 genes associated with carbohydrate transport and metabolism, which constituted 6.47%. Furthermore, 111 genes were linked to the biosynthesis, transport, and breakdown of secondary metabolites, representing 2.82% of the annotated genes (Figure 2B).

Upon integrating annotations from KEGG, GO, and COG, M4 demonstrated broad functionality and high adaptability across multiple biological processes. These functional genes probably give M4 a competitive advantage in complex environments, especially in nutrient use, metabolic regulation, and stress responses [18,27].

### 3.4. Identification of the NK-Encoding Gene

**Figure 4:**
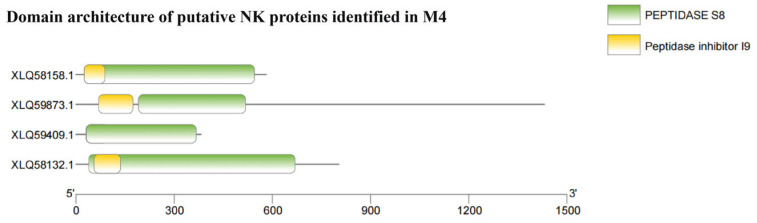
Identification of candidate NK proteins in *B. velezensis* M4.

NK is a serine protease with potent thrombolytic activity, primarily produced by bacteria during natto fermentation. This enzyme hydrolyzes fibrin and belongs to the subtilisin-like serine protease family. Its precursor typically contains two characteristic domains: the peptidase inhibitor I9 (pfam05922) propeptide domain and the peptidase S8 (pfam00082) catalytic domain. The I9 domain facilitates correct protein folding and maintains proenzyme inhibition, whereas the S8 domain mediates the catalytic activity of the mature enzyme. We previously determined that the fermentation supernatant of strain M4 exhibited a NK activity of 318.96 U/g, indicating considerable fibrinolytic capacity. To elucidate the molecular basis of this activity, we performed domain annotation analysis on the predicted proteins from the M4 genome. Four proteins (XLQ58132.1, XLQ58158.1, XLQ59409.1, and XLQ59873.1) were found to contain both I9 and S8 domains, an architecture consistent with typical NK (Figure 4). The integration of enzyme activity data and domain annotation suggests that the observed fibrinolytic activity likely stems from one or more of these proteases. However, definitive confirmation of their identity and functional contribution requires further experimental validation, including sequence alignment, gene knockout, protein purification coupled with biochemical characterization, or heterologous expression.

### 3.5. Analysis of CAZymes, Secondary Metabolites, and Proteolytic Enzymes

The genome of M4 encoded 132 putative CAZymes, which were classified into six major categories: 42 glycosyl transferases (GTs), 4 auxiliary activities (AAs), 3 polysaccharide lyases (PLs), 12 carbohydrate esterases (CEs), 48 glycoside hydrolases (GHs), and 23 carbohydrate-binding modules (CBMs) (Table S4). The most abundant family was GH, accounting for 36.36% of all CAZyme genes, followed by GT (31.82%), CBM (17.42%), CE (9.09%), AA (3.03%), and PL (2.27%). Several families—such as GH18, GH23, CE4, and GH171—are involved in the breakdown of peptidoglycan [22]. Furthermore, GH18, GH23, and GH73 are associated with lysozyme-like activities [8,32]. CBM50, known to facilitate the targeting and binding of certain antimicrobial enzymes or proteins, can be attached to various GH family enzymes to hydrolyze peptidoglycan, thereby disrupting bacterial cell wall integrity [19,33]. These findings suggest that M4 exerts its antibacterial effects via peptidoglycan degradation and lysozyme-like activity [34], emphasizing the crucial role of CAZyme genes in its antimicrobial function.

As listed in Table 2, of the various secondary metabolites, six compounds demonstrated antimicrobial activity: macrolactin H, bacillaene, fengycin, difficidin, bacillibactin, and bacilysin. Furthermore, M4 was predicted to produce three secondary metabolites with different degrees of similarity to plantazolicin (91%), surfactin (86%), and butirosin A/butirosin B (7%). For two terpene clusters, one T3PKS, one phosphonate, and one NRPS, similar compounds could not be identified from the database, which implies that they are unknown. The fact that M4 encodes various antimicrobial compounds, including polyketides, antibiotics, antimicrobial peptides, and lipopeptides, suggests that the strain uses these compounds to exert its antimicrobial activity.

Of all the isolates tested, strain M4 displayed the highest protease activity. Genomic analysis revealed that M4 contained 142 proteolytic enzymes, highlighting its strong proteolytic capabilities. The main protease families identified were M20, C26, S08, S09, C40, S14, and G02 (Supplementary Material S4). Of these, the M20 family, which includes aminopeptidases, was the most prevalent. M20 enzymes function in the final stage of protein degradation and are essential for the utilization of exogenous proteins and the recycling of intracellular proteins [35].

### 3.6. Comparative Genome Analysis

**Figure 5:**
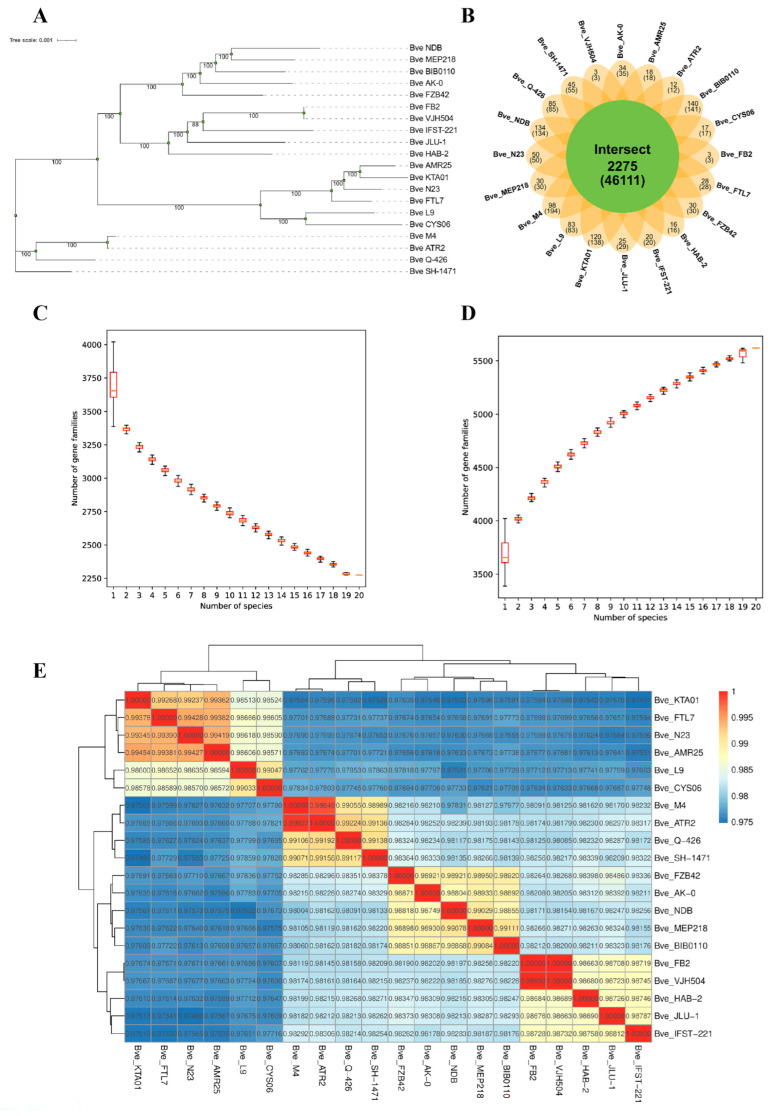
Comparative genomic analysis of *B. velezensis*. (**A**) Phylogenetic tree; (**B**) Venn diagram of homologous gene families. Each ellipse represents a genome, and the numbers above each region indicate the number of gene families in the species of that region. The numbers in parentheses below indicate the total number of genes in the gene families of the species in that region; (**C**) Core gene box plot; (**D**) Pangenome box plot. (**E**) The ANI values (%) between *B. velezensis* M4 and the other 19 strains of *B. velezensis*.

Nineteen genomes of *B. velezensis* were chosen from the NCBI database for comparative genome analysis. Phylogenetic analysis confirmed that M4 belonged to *B. velezensis*. The phylogenetic tree indicated that M4 was evolutionarily closest to *B. velezensis* ATR2 (Figure 5A) and also closely related to *B. velezensis* Q426, while exhibiting significant genetic differences from the other 17 strains. As shown in Figure 5E, the ANI value was 99.9% between M4 and ATR2, followed by Q426 and SH-1471 at 99.10% and 99.07%, respectively. Supplementary Material S2 reports an AAI of 99.94% between M4 and ATR2 and 99.05% with Q-426.

*B. velezensis* ATR2, isolated from ginger’s rhizosphere, is beneficial for preventing and controlling ginger rhizome rot. Its secondary metabolites include three cyclic lipopeptides (surfactin, bacillomycin D, and fengycin), three polyketides (macrolactin, difficidin, and bacillaene), and one dipeptide (bacilysin). Furthermore, *B. velezensis* ATR2 possesses a broad antimicrobial spectrum, effectively inhibiting the growth of *E. coli*, *S. aureus*, and *Salmonella*. Moreover, it demonstrates activity against various plant pathogens, inhibiting the mycelial growth of *Fusarium graminearum* Sehw, *Botrytis cinerea*, and *Ceratocystis paradoxa* (Dode) Moreau [36]. *B. velezensis* Q-426 also synthesizes secondary metabolites that display antimicrobial properties. Wang et al. successfully identified 12 lipopeptides, including surfactin, iturin, and fengycin, in the fermentation broth of *B. velezensis* Q-426 [37]. Our previous study found that M4 encodes a range of antimicrobial compounds, including polyketides, antibiotics, antimicrobial peptides, and lipopeptides. This finding signifies that, similar to *B. velezensis* ATR2, these substances likely contribute to M4’s antimicrobial properties. Pangenomic analysis is often used to explore the genetic diversity among microorganisms [38]. As illustrated in Figure 5C,D, increasing the number of genomes decreased the number of core genes, whereas the total gene count in the pangenome increased. This observation suggests that *B. velezensis* has a large and open pangenome [22]. During the genomic evolution of a species, a gene can duplicate, creating multiple copies that form homologous gene families. These homologous genes share similar structures and functions. In Figure 5B, each ellipse depicts a genome, and the numbers above each region indicate the number of gene families in the species [39]. The numbers in parentheses denote the total number of genes in each gene family for the species in that region. The center of the figure shows homologous genes shared across all strains. The results revealed that M4 and the 19 other strains shared 2275 homologous gene families. Additionally, M4 harbored 98 unique gene families, which may confer strain-specific functions and account for its distinct characteristics.

Collinearity refers to a situation in which specific chromosomal regions in different species exhibit similar gene arrangements. Generally, the greater the evolutionary distance between species, the less gene collinearity they tend to show [40]. Therefore, the level of collinearity between two species can indicate their evolutionary distance. In this study, *B. velezensis* ATR2 and *B. velezensis* L9 were selected as reference genomes, and collinearity analysis was used to compare the evolutionary distance between M4 and these two species. As depicted in Figure S2 (Supplementary Material S1), M4 demonstrated strong collinearity with *B. velezensis* ATR2 but weak collinearity with *B. velezensis* L9. Furthermore, collinearity analysis can identify structural variations in genomes that occurred over evolution. For instance, Supplementary Materials S3 and S4 reveal that the genomes of M4 and *B. velezensis* ATR2 contain single-base substitutions, transversions, and long-segment sequence insertions, deletions, inversions, and translocations over 50 bp long. These phenomena may explain why M4 possesses certain unique genes.

### 3.7. Safety Evaluation of M4

**Figure 6:**
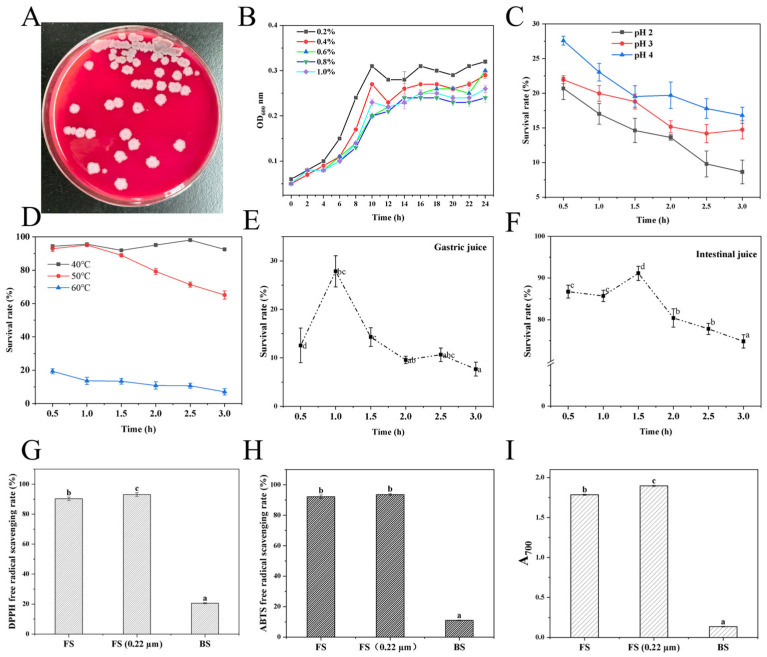
Hemolytic activity and probiotic properties of *B. velezensis* M4. (**A**) Hemolytic activity; (**B**) Bile salt tolerance; (**C**) pH tolerance; (**D**) Temperature tolerance; (**E**) Gastric juice tolerance; (**F**) Intestinal fluid tolerance; (**G**) DPPH-radical scavenging rate; (**H**) ABTS-radical scavenging rate; (**I**) FRAP assay. Different lowercase superscript letters (a–d) in sub-figures (**E**–**I**) indicate significant differences among treatments according to Duncan’s multiple range test (*p* < 0.05).

Figure 6A showed that M4 did not cause hemolysis, highlighting its γ-hemolytic nature and confirming its nontoxicity. The lack of hemolysis is a vital initial screening criterion for potential probiotic candidates. Additionally, given the rising antibiotic resistance among microbes owing to misuse, assessing the antibiotic resistance profile is crucial for both safety evaluation and biosafety assurance of probiotics. In this study, M4’s susceptibility to 16 commonly used antibiotics was tested. As presented in Table S5, M4 was susceptible to chloramphenicol, tetracycline, cefotaxime, amikacin, florfenicol, ciprofloxacin, rifampicin, and minocycline. Genome analysis using AMRFinderPlus v4.0.23 identified four antibiotic resistance genes: *ant (6)*, *bla*, *tet*, and *satA* (Supplementary Material S4), suggesting potential resistance to aminoglycosides, β-lactams, tetracyclines, and streptothricin [41,42]. However, the in vitro susceptibility results did not fully align with these genomic predictions. For example, M4 exhibited high tetracycline susceptibility despite carrying *tet*. This difference implies that carrying resistance genes in the genome does not always result in their phenotypic expression [28]. Meanwhile, it should be emphasized that the presence of these ARGs does not directly equate to clinically relevant multidrug resistance or resistance to all related antibiotics. The actual resistance phenotype is also influenced by multiple factors, including gene expression regulation, mutations, and the physiological state of the bacterium.

A total of 631 VF-related proteins were identified in strain M4. It should be emphasized that the vast majority of these proteins are not classical virulence determinants, but rather housekeeping or environmental adaptation genes commonly present in the genus *Bacillus*. The main functional categories included immune modulation (173), nutritional metabolism (150), motility (73), regulation (51), and exotoxins (50), with the most frequently annotated genes being pks (16) and flmH (13) (Supplementary Material S4). Comparative genomic analysis revealed that *B. velezensis* as a species typically harbors numerous VFDB-annotated homologs, indicating that this VF profile is not strain-specific. Notably, in non-pathogenic bacteria such as *B. velezensis*, these VFs are more likely involved in ecological adaptation, interspecies competition, host (e.g., plant) interactions, and biofilm formation, rather than directly indicating pathogenicity toward mammals. Collectively, the VF repertoire of M4 reflects the characteristic environmental adaptation and interaction features of *B. velezensis*, potentially supporting complex interactions with substrates and other microbes in fermentation environments [18,43,44,45]. Nevertheless, given the tendency of VFDB to over-annotate non-pathogenic bacteria, transcriptomic and proteomic analyses, together with in vivo safety assessments (e.g., acute toxicity tests), are required to validate the actual functions of these genes and rigorously evaluate the safety of M4 as a probiotic candidate.

### 3.8. Probiotic Evaluation of Strain M4

M4 exhibited a survival rate of 13.62% after 2 h at pH 2.0, indicating limited acid tolerance. Although the majority of cells were inactivated under this extreme condition (Figure 6C), a viable subpopulation persisted. In contrast, the strain displayed pronounced thermotolerance at 50 °C and 60 °C, with 65.14% survival after 3 h at 50 °C (Figure 6D). Genomic analysis revealed potential genetic determinants of acid resistance, including genes encoding Na^+^/H^+^ antiporters (*mrp/mnh/sha*, *nhaA/B/C*) and F_0_F_1_-ATP synthase (*atpA~I*). Similarly, heat resistance might be associated with molecular chaperones and regulatory factors (*dnaK/dnaJ/grpE*, *groEL/groES*, *ctsR*, *clpP/clpC/clpE/clpX*, and *cspA/B/C/D*) (Figure 2A) [46,47]. However, the actual expression and functional contributions of these genes under the tested conditions require validation via transcriptomic or proteomic analyses.

Figure 6B illustrated the growth curves of M4 under bile salt stress (0.2–1.0%). The strain maintained high viability at 0.2% and 0.4% bile salt concentrations. KEGG annotation identified genes encoding a putative bile salt hydrolase, potentially contributing to bile salt resistance through detoxification. However, direct evidence of gene expression is required to confirm this function. Furthermore, M4 demonstrated robust survival under simulated gastrointestinal conditions. Following 1 h exposure to simulated gastric juice, the survival rate was 27.85%, with persistent viability at 3 h (Figure 6E). The strain also exhibited marked tolerance to simulated intestinal fluid, retaining 74.80% survival after 3 h (Figure 6F). Probiotics typically benefit from the capacity to tolerate gastric acidity and intestinal bile salts to successfully reach and colonize target sites [46]. Collectively, the acid and bile salt resilience of M4 indicates potential for gastrointestinal transit survival, though in vivo studies are needed to validate its probiotic efficacy.

Certain probiotics confer health benefits by producing antioxidant compounds in the gut, thereby maintaining redox homeostasis and mitigating oxidative damage associated with aging and chronic diseases [47]. To evaluate this potential, the in vitro antioxidant activity of M4 fermentation supernatant (FS) and bacterial suspension (BS) was assessed (Figure 6G–I). The supernatant demonstrated potent antioxidant capacity, with DPPH and ABTS radical scavenging rates of 90.25% ± 1.16% and 92.16% ± 1.15%, respectively, alongside high total reducing power. Following membrane filtration, DPPH scavenging activity increased to 93.06% ± 1.22% (*p* < 0.05), whereas ABTS activity showed no significant change (93.59% ± 0.72%, *p* > 0.05); total reducing power was significantly enhanced (*p* < 0.05). Genomic analysis revealed that M4 harbors numerous genes implicated in antioxidant defense, including catalases (*katA/E/X*), superoxide dismutase (*sodA*), and components of the peroxiredoxin (*ahpC/F*), organic hydroperoxide resistance (*ohrA/R*), and thioredoxin (*trxA/B*, *tpx*) systems, among others (*msrA/B*, *bshA/B/C*, *perR*, *dps*) (Figure 2A) [28,30]. The presence of these genes suggests a robust antioxidant and stress response system that may protect cellular integrity during oxidative challenge. This genetic repertoire aligns with the observed high antioxidant activity of the fermentation products. Nevertheless, direct enzymatic assays and gene expression analyses (e.g., qPCR, RNA-seq) are required to establish a causal relationship between genotype and antioxidant phenotype.

**Figure 7:**
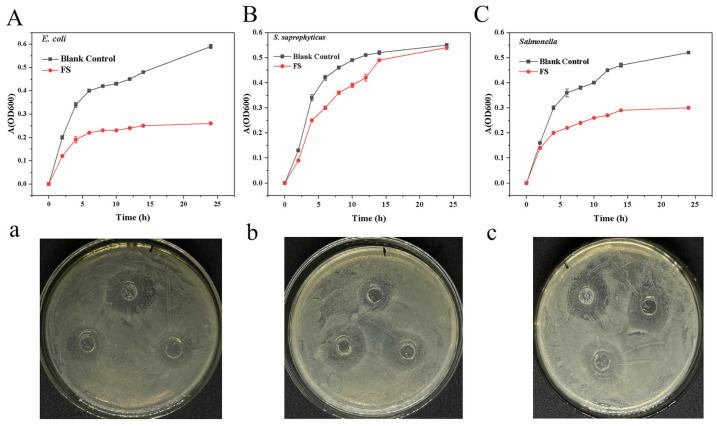
Antibacterial activity of *B. velezensis* M4. (**A**,**a**) Inhibitory effect on *E. coli*; (**B**,**b**) Inhibitory effect on *S. aureus*; (**C**,**c**) Inhibitory effect on *Salmonella*. All experiments were performed in triplicate, and data are expressed as mean ± standard deviation (SD). Statistical analysis was conducted using Duncan’s multiple range test, with a significance threshold of *p* < 0.05.

Figure 7 illustrated the inhibitory effects of FS against *E. coli*, *S. saprophyticus*, and *Salmonella* spp. Compared with the blank control, the supernatant suppressed growth of all three target bacteria, with inhibition rates of 38.49%, 13.95%, and 32.56% at 12 h, respectively. Antimicrobial activity was further evaluated by disk diffusion assay, yielding inhibition zones of 16.80 ± 0.61 mm, 14.24 ± 1.92 mm, and 16.30 ± 1.62 mm. These effects may be attributed to antimicrobial proteins and lipopeptides present in the supernatant. Genomic analysis revealed 37 BGCs in strain M4, spanning 12 categories predominantly for nonribosomal peptides and polyketides (Supplementary Material S1: Figure S1 and Supplementary Material S4. As detailed in Table 2, M4 was predicted to produce several antimicrobial compounds, including macrolactin H, bacillaene, fengycin, difficidin, bacillibactin, and bacilysin. Notably, five BGCs—two terpene clusters, one T3PKS, one phosphonate, and one NRPS—showed no homology to known compounds, suggesting the potential for novel antimicrobials. This diversity of antimicrobial-related BGCs indicates that M4 may combat other microbes through multiple mechanisms [8,34]. However, actual production of these compounds under specific fermentation conditions requires verification by LC-MS, and the contribution of individual BGCs to the observed antibacterial activity warrants investigation via gene knockout or heterologous expression studies.

## 4. Conclusions

In this study, *B. velezensis* M4, exhibiting high nattokinase activity and broad-spectrum antimicrobial activity, was isolated from traditional fermented soybean paste in Northeast China. Whole-genome sequencing revealed a 4,473,838 bp genome with 46.94% GC content, encoding 4516 predicted proteins. Functional annotation identified four proteins containing characteristic NK domains (I9 and S8), as well as 37 biosynthetic gene clusters potentially producing six antimicrobial secondary metabolites. Safety assessment demonstrated absence of hemolytic activity, susceptibility to multiple antibiotics, and lack of high-risk virulence factor genes. In vitro probiotic characterization revealed that the strain exhibited a certain degree of gastrointestinal tolerance, acid resistance, and bile salt tolerance, along with favorable antioxidant and antibacterial capacities.

However, this study is subject to several limitations. The primary constraint lies in the exclusively in vitro nature of functional assessments, rendering the in vivo efficacy of M4 yet to be established. Equally important, functional genes predicted from the genome—including those encoding NK and secondary metabolite biosynthetic clusters—remain unvalidated at the transcriptional or protein level, highlighting the discrepancy between gene presence and functional expression. Furthermore, although 98 strain-specific genes were identified in the genome of M4 through comparative genomic analysis, their functional relevance has not been explored in this study, which represents a limitation. Consequently, future studies should prioritize animal experiments to evaluate thrombolytic activity and gut microbiota-modulating capacity, complemented by transcriptomic, proteomic, gene knockout, or heterologous expression approaches to systematically dissect the molecular mechanisms underlying these functional traits. Specifically, functional characterization of the strain-specific genes is warranted to better understand the unique advantages of M4.

## Figures and Tables

**Table 1:** Morphological and physiological-biochemical characteristics of strain M4.

Identification Index	Result	Identification Index	Result
Gram staining	Positive	Citrate utilization	−
Cell morphology	Short rod	Starch hydrolysis	+
Endospore staining	+	Casein hydrolysis	+
pH	6.94	Catalase test	+
14% NaCl	+	Glucose fermentation	+
NK activity	318.96 U/g	Mannitol fermentation	+
Cellulase activity	52.63 ± 0.77 U/mg	Rhamnose fermentation	−
α-Amylase activity	5.60 ± 0.23 U/mg	Voges-Proskauer (V.P) test	+
		Phenylalanine deaminase test	−

Note: “+” (Positive): Indicates the presence of a specific characteristic or reaction. “−” (Negative): Indicates the absence of a specific characteristic or reaction.

**Table 2:** Identification of the region of secondary metabolite synthesis in M4.

Cluster ID	Initial Site	Terminal Site	Product Type	Most Similar Product	Similarity, %
1	312,307	377,698	NRPS	surfactin	86%
2	635,917	648,061	phosphonate	-	-
3	706,825	730,002	RRE-containing, LAP	plantazolicin	91%
4	1,016,745	1,057,989	PKS-like	butirosin A/butirosin B	7%
5	1,140,687	1,161,427	terpene	-	-
6	1,472,617	1,560,847	transAT-PKS	macrolactin H	100%
7	1,785,765	1,895,864	transAT-PKS, T3PKS, NRPS	bacillaene	100%
8	2,058,550	2,196,382	NRPS, betalactone, transAT-PKS	fengycin	100%
9	2,245,565	2,267,448	terpene	-	-
10	2,349,052	2,390,152	T3PKS	-	-
11	2,504,791	2,610,964	transAT-PKS	difficidin	100%
12	3,444,744	3,496,537	NRP-metallophore, NRPS, RiPP-like	bacillibactin	100%
13	3,777,141	3,845,561	NRPS	-	-
14	4,119,000	4,160,418	other	bacilysin	100%

## Data Availability

The original contributions presented in this study are included in the article/Supplementary Materials. Further inquiries can be directed to the corresponding author.

## Supplementary Materials

The following supporting information can be downloaded at: https://www.mdpi.com/article/10.3390/foods15091553/s1, Figure S1: Secondary metabolite biosynthetic gene cluster prediction; Figure S2: The collinearity relationship between *B. velezensis* M4 and *B. velezensis* ATR2 (A), as well as the collinearity relationship between *B. velezensis* M4 and *B. velezensis* L9 (B). Table S1: Strain screening results. Table S2: Statistics of open reading frame (ORF) predictions; Table S3: Statistics of non-coding RNA predictions. Table S4: Carbohydrate-active enzymes analysis statistics. Table S5: Criteria for determination of drug sensitive paper. Table S6: Source and genomic information of 19 *B. velezensis* strains.

## Abbreviations

The following abbreviations are used in this manuscript:

BGCs	Biosynthetic gene clusters
NK	Nattokinase
VFs	Virulence factors
VFD	Virulence factors database
COG	Clusters of orthologous groups
CDS	Coding DNA sequence
KEGG	Kyoto Encyclopedia of Genes and Genomes
GO	Gene Ontology
CAZYme	Carbohydrate-active enzyme
SNP	Single-nucleotide polymorphism
SV	Structural variation
InDel	Insertion–Deletion
ANI	Average nucleotide identity
AAI	Amino acid identity
