# Supplementary material for: Bacillus velezensis M4 from Northeast Chinese Soybean Paste Combines Nattokinase and Antibacterial Activities
Source: Foods. 2026 Apr 30;15(9):1553. doi: 10.3390/foods15091553 (PMC13164281; doi:10.3390/foods15091553)
Supplement: Supplementary file 1 [file foods-15-01553-s001.zip › foods-4247750-supplementary/Supplementary Material S1.pdf]

Supplementary Material

Table S1 Strain screening results

Strain No.	Colony characteristics	Cell morphology	Inhibition zone	NK activity (U/mL)
B1	dry and wrinkled	long rod-shaped	-	90.0
B2	Slightly moist, wrinkled	short rod-shaped	-	300.0
B3	Dry, raised	long rod-shaped	-	172.0
B4	Concave and sticky	short rod-shaped	-	113.0
B5	Dry, wrinkled	short rod-shaped	-	214.0
M1	Concave, raised, and sticky	short rod-shaped	-	134.0
M2	Dry, slightly raised	short rod-shaped	-	78.0
M3	Concave, raised, and sticky	short rod-shaped	-	88.0
M4	Concave, raised, and sticky	short rod-shaped	+	319.0
M5	Concave, raised, and sticky	long rod-shaped	-	205.0
H1	Concave, raised, and sticky	long rod-shaped	-	251.0
H2	Concave, raised, and sticky	short rod-shaped	-	130.0
H3	Concave, raised, and sticky	short rod-shaped	-	89.0
H4	Concave, raised, and sticky	short rod-shaped	-	136.0
H5	Concave, raised, and sticky	short rod-shaped	-	143.0
C1	Concave, raised, and sticky	short rod-shaped	-	216.0
C2	Concave, raised, and sticky	short rod-shaped	-	137.0
C3	Concave, raised, and sticky	short rod-shaped	-	225.0
C4	Concave, raised, and sticky	short rod-shaped	-	217.0
C5	Concave, raised, and sticky	short rod-shaped	-	265.0

Note: "+" indicates the presence of an inhibition zone, and "-" indicates the absence of an inhibition zone.

Table S2 Statistics of open reading frame (ORF) predictions

ORF num	4,516
ORF total length	3,960,789 bp
ORF density	1009.42 genes per kb
Longest ORF length	17,103 bp
ORF average length	878.67 bp
Intergenetic region length	513,049 bp
ORF/Genome(coding percentage)	88.53%
Intergenetic length/Genome	11.47%
GC content in ORF region	46.94%
GC content in intergenetic region	39.87%

Table S3 Statistics of non-coding RNA predictions

Type	Copy Number	Avg. length (bp)	Total length (bp)	percent of genome (%)
5S rRNA	9	111	999	0.0223
16S rRNA	9	1547.22	13925	0.3112
23S rRNA	9	2926	26334	0.5886
tRNA	86	77.17	6637	0.1483
nc RNA	93	155.59	14470	0.3234

Table S4 Carbohydrate-active enzymes analysis statistics

Type	Number of Genes	Percentage (%)
Glycoside hydrolases (GHs)	48	36.36
Glycosyl transferases (GTs)	42	31.82
Carbohydrates (CEs)	12	9.09
Auxiliary activities (AAs)	4	3.03
Polysaccharides (PLs)	3	2.27
Carbohydrate-Binding Module (CBM)	23	17.42

Table S5 Criteria for determination of drug sensitive paper

Antibiotics	Drug Content (μg/piece)	Inhibitory diameter (mm)	Antibiotic susceptibility of the strain
Erythromycin	15	0±0	R
Chloramphenicol	30	22.44±1.16	S
Tetracycline	20	21.52±1.35	S
Vancomycin	30	18.14±1.46	S
Kanamycin	30	8.6±0.4	R
Clindamycin	2	0±0	R
Streptomycin	10	14.4±0.4	I
Gentamicin	10	9.2±0.51	R
Ampicillin	20	14.02±0.44	R
Cefotaxime	30	21.19±0.41	S
Amikacin	30	15.62±0.29	S
Florfenicol	30	24.7±0.74	S
Ciprofloxacin	5	17.46±0.92	I
Rifampicin	5	27.81±0.84	S
Lincomycin	2	0±0	R
Minocycline	30	22.4±0.78	S

S = Susceptible   I = Intermediate   R = Resistant

Table S6 Source and Genomic Information of 19 *Bacillus velezensis* Strains

Species Name	GenBank	Reported in Literature	Journal Name
Bacillus velezensis FTL7	GCA_022816 825.1	Complete genome sequencing and strain characterization of a novel marine *Bacillus velezensis* FTL7 with a potential broad inhibitory spectrum against foodborne pathogens	World Journal of Microbiology and Biotechnology
Bacillus velezensis HAB-2	GCA_014211 995.1	Comparative Genomics Analysis Provides New Strategies for Bacteriostatic Ability of *Bacillus velezensis* HAB-2	Frontiers in Microbiology
Bacillus velezensis NDB	GCA_032254 155.1	Analysis of antimicrobial biological activity of a marine *Bacillus velezensis* NDB	Archives of Microbiology
Bacillus velezensis ATR2	GCA_002761 535.1	Genomic, Antimicrobial, and Aphicidal Traits of *Bacillus velezensis* ATR2, and Its Biocontrol Potential against Ginger Rhizome Rot Disease Caused by *Bacillus* pumilus	Microorganisms
Bacillus velezensis AMR25	GCA_035340 015.1	Whole Genome Sequencing of *Bacillus velezensis* AMR25, an Effective Antagonist Strain against Plant Pathogens	Microorganisms
Bacillus velezensis JLU-1	GCA_040571 435.1	Antagonistic Mechanism Analysis of *Bacillus velezensis* JLU-1, a Biocontrol Agent of Rice Pathogen Magnaporthe oryzae	Journal of Agricultural and Food Chemistry
Bacillus velezensis N23	GCA_033547 015.1	Genome Sequencing and Characterization of *Bacillus velezensis* N23 as Biocontrol Agent against Plant Pathogens	Microorganisms
Bacillus velezensis SH-1471	GCA_030378 485.1	Whole genome sequencing provides evidence for *Bacillus velezensis* SH-1471 as a beneficial rhizosphere bacterium in plants	Scientific Reports
Bacillus velezensis AK-0	GCA_014706 595.1	Characterization of *Bacillus velezensis* AK-0 as a biocontrol agent against apple bitter rot caused by Colletotrichum gloeosporioides	Scientific reports
B. velezensis IFST-221	GCA_030028 255.1	Identification and genomic insights into a strain of *Bacillus velezensis* with phytopathogen-inhibiting and plant growth-promoting properties	Microbiological Research
Bacillus velezensis L9	GCA_033022 835.1	A Novel *Bacillus Velezensis* for Efficient Degradation of Zearalenone	Foods
Bacillus velezensis	GCA_033170	The *Bacillus velezensis* CYS06	Fishes

Species Name	GenBank	Reported in Literature	Journal Name
CYS06	215.1	Strain Exhibits Promising Applications in Fighting Grass Carp Bacterial Diseases	
Bacillus velezensis VJH504	GCA_030704 265.1	Genome sequencing and analysis of *Bacillus velezensis* VJH504 reveal biocontrol mechanism against cucumber Fusarium wilt	Frontiers in Microbiology
Bacillus velezensis FZB42	GCA_000015 785.2	High-Efficiency CRISPR-Cas9 Genome Editing Unveils Biofilm Insights and Enhances Antimicrobial Activity in *Bacillus velezensis* FZB42	Biotechnology and Bioengineering
Bacillus velezensis MEP218	GCA_023612 895.2	Unraveling the genome of Bacillus velezensis MEP218, a strain producing fengycin homologs with broad antibacterial activity: comprehensive comparative genome analysis	Scientific Reports
Bacillus velezensis KTA01	GCA_029590 295.1	Biocontrol of Peach Gummosis by Bacillus velezensis KTA01 and Its Antifungal Mechanism	Journal of Microbiology and Biotechnology
Bacillus velezensis FB2	GCA_025290 695.1	Food safety and biological control; genomic insights and antimicrobial potential of Bacillus velezensis FB2 against agricultural fungal pathogens	PLOS ONE
Bacillus velezensis Q-426	GCA_024610 675.1	Genomic and metabolomic insights into the antimicrobial compounds and plant growth-promoting potential of *Bacillus velezensis* Q-426	BMC Genomics
Bacillus velezensis BIB0110	GCA_025753 225.1	Draft Genome Sequence of the Plant Growth Promoter and Biocontrol Agent *Bacillus velezensis* Strain BIB0110	Microbiology Resource Announcements

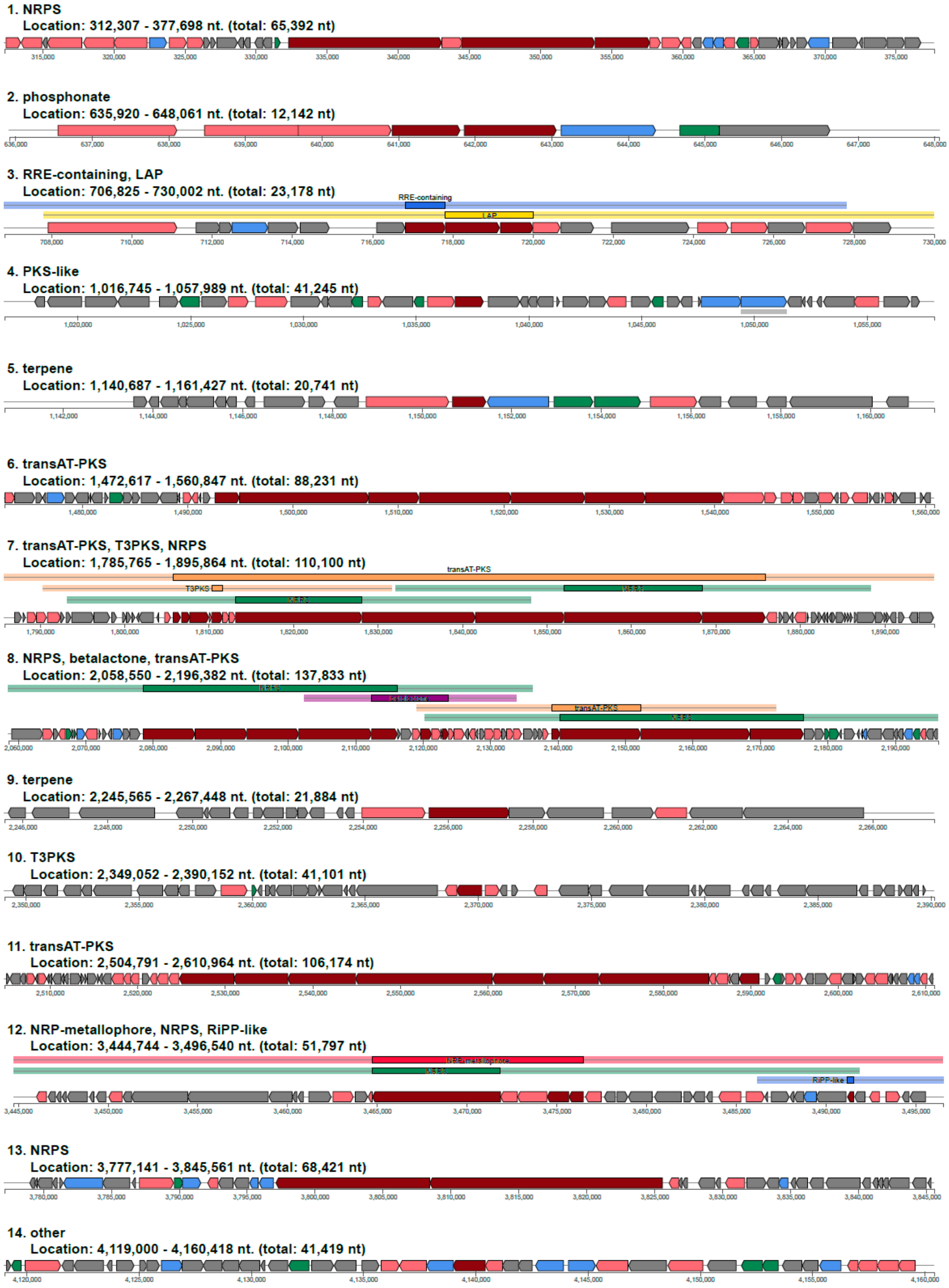

Figure S1 Secondary metabolite biosynthetic gene cluster prediction

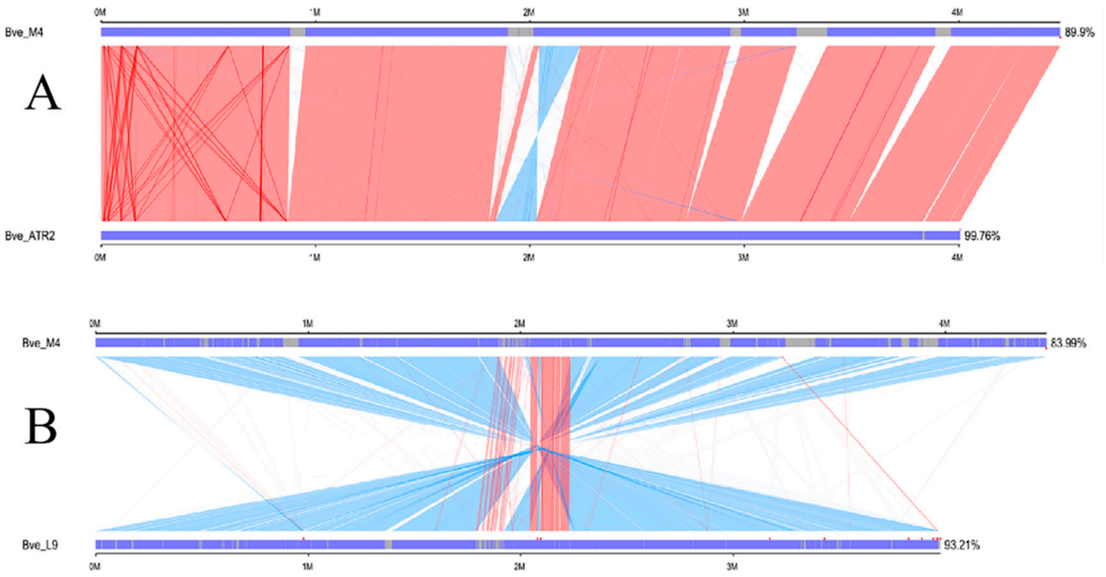

Figure S2 The collinearity relationship between *B. velezensis* M4 and *B. velezensis* ATR2 (A), as well as the collinearity relationship between *B. velezensis* M4 and *B. velezensis* L9 (B).
